# Contemporary Insights into Hepatitis C Virus: A Comprehensive Review

**DOI:** 10.3390/microorganisms12061035

**Published:** 2024-05-21

**Authors:** Malik Sallam, Roaa Khalil

**Affiliations:** 1 Department of Pathology, Microbiology and Forensic Medicine, School of Medicine, The University of Jordan, Amman 11942, Jordan; 2 Department of Clinical Laboratories and Forensic Medicine, Jordan University Hospital, Amman 11942, Jordan

**Keywords:** HCV, viral hepatitis, virology, hepatology, genotype, drug resistance, prevention

## Abstract

Hepatitis C virus (HCV) remains a significant global health challenge. Approximately 50 million people were living with chronic hepatitis C based on the World Health Organization as of 2024, contributing extensively to global morbidity and mortality. The advent and approval of several direct-acting antiviral (DAA) regimens significantly improved HCV treatment, offering potentially high rates of cure for chronic hepatitis C. However, the promising aim of eventual HCV eradication remains challenging. Key challenges include the variability in DAA access across different regions, slightly variable response rates to DAAs across diverse patient populations and HCV genotypes/subtypes, and the emergence of resistance-associated substitutions (RASs), potentially conferring resistance to DAAs. Therefore, periodic reassessment of current HCV knowledge is needed. An up-to-date review on HCV is also necessitated based on the observed shifts in HCV epidemiological trends, continuous development and approval of therapeutic strategies, and changes in public health policies. Thus, the current comprehensive review aimed to integrate the latest knowledge on the epidemiology, pathophysiology, diagnostic approaches, treatment options and preventive strategies for HCV, with a particular focus on the current challenges associated with RASs and ongoing efforts in vaccine development. This review sought to provide healthcare professionals, researchers, and policymakers with the necessary insights to address the HCV burden more effectively. We aimed to highlight the progress made in managing and preventing HCV infection and to highlight the persistent barriers challenging the prevention of HCV infection. The overarching goal was to align with global health objectives towards reducing the burden of chronic hepatitis, aiming for its eventual elimination as a public health threat by 2030.

## 1. Introduction

Chronic hepatitis C remains a significant global health burden affecting a substantial number of individuals worldwide [1,2,3]. Based on the World Health Organization (WHO) estimates, there were about 50 million people living with chronic hepatitis C by the year 2023 worldwide, with an estimated 1 million new infections each year [3,4]. Additionally, about 3.26 million children were affected by chronic hepatitis C, highlighting its significant burden [5,6].

Following the first decade of the new millennium, the availability and approval of direct-acting antivirals (DAAs) marked a new era in the management of chronic hepatitis C [7,8]. The availability of these oral curative agents represented the culmination of over three decades of intensive research [9]. The DAAs are characterized by remarkable safety and effectiveness profiles, leading to achievement of sustained virologic response (SVR)—which marks the cure—in more than 90% of individuals with chronic hepatitis C [10,11,12].

The current approved therapeutic protocols for the treatment of chronic hepatitis C virus (HCV) infection issued by the Infectious Diseases Society of America (IDSA) and the American Association for the Study of Liver Diseases (AASLD)—titled “the HCV guidance”—involve combining various DAAs [13,14]. These DAAs can be classified as NS3 protease inhibitors (PIs, with the drug names having the suffix -previr), NS5A replication complex inhibitors (NS5AIs, with the drug names having the suffix -asvir), and NS5B polymerase nucleoside (NI) and non-nucleoside (NNI) inhibitors (with the drug names having the suffix -buvir) [15,16].

The first generation of DAAs comprises (1) NS5AIs including Daclatasvir, Ledipasvir, Elbasvir, and Ombitasvir; (2) PIs including Simeprevir, Grazoprevir, Asunaprevir, and Paritaprevir; and (3) the NNI represented by Dasabuvir [17,18]. The first-generation agents exhibit genotype-specific activity [19,20]. In contrast, the second-generation DAAs exhibit pan-genotypic activity with notable efficacy, including the NS5AIs Pibrentasvir and Velpatasvir, the PIs Glecaprevir and Voxilaprevir, and the NI Sofosbuvir [17,20].

Regarding the prevention of HCV and its possible eradication, significant challenges remain [21,22]. These challenges stem from the extensive genetic variability of HCV and the challenge of eliciting a protective immune response against the virus, with both factors representing a major obstacle to the development of an effective vaccine [23,24,25]. Nevertheless, optimism grew regarding the prospects of HCV elimination globally with the availability and success of DAAs as a curative strategy [16,26,27,28]. Thus, the promising goal of eliminating HCV as a public health threat relies on the “Treatment as Prevention” (TasP) strategy [29,30,31].

A major obstacle to the sustained success of DAAs as a curative treatment for HCV, let alone HCV eradication, is the emergence of HCV variants resistant to DAAs [32,33,34,35]. The presence of resistance-associated substitutions (RASs), which can occur naturally or develop during treatment, has the potential to reduce the effectiveness of treatment, with the possibility of forward transmission of drug-resistant variants, especially within high-risk populations [36,37,38].

Given the disparities in DAA access especially in low-income settings, variations in treatment responses among diverse populations, and the rise of RASs, a contemporary reassessment of HCV management is necessary. Additionally, the evolving epidemiological trends, ongoing advances in therapeutic modalities, and challenges in HCV prevention by vaccination necessitate an updated comprehensive review. Therefore, the aim of this comprehensive review was to provide an up-to-date source of information regarding HCV, including aspects of epidemiology, clinical features, diagnosis, treatment, and prevention, with a focus on addressing the challenges posed by DAA resistance and vaccine development efforts.

## 2. History of Hepatitis C

It became evident by the mid-1970s that a viral hepatitis agent, distinct from hepatitis A virus (HAV) and hepatitis B virus (HBV), was responsible for post-transfusion hepatitis, and this putative clinical entity was termed “non-A, non-B” hepatitis (NANBH) [16,39,40].

Subsequent investigation involving transfusion recipients revealed that acute clinical manifestations of NANBH were generally less severe than those of hepatitis; nevertheless, NANBH was capable of leading to grave outcomes, including cirrhosis and hepatic failure [41]. Experiments involving the inoculation of chimpanzees with blood from subjects afflicted by acute and chronic NANBH demonstrated a notable rise in hepatic transaminase; thus, these chimpanzees provided a crucial animal model to understand the nature of NANBH chronicity [42,43]. Subsequent investigations helped to characterize the nature of the NANBH culprit agent as a small, enveloped virus of less than 80 nm in diameter [44,45,46]; nevertheless, this agent was elusive to traditional viral culture and immunological identification techniques [47,48]. Progress was made through the serial passage of NANBH in chimpanzees, which provided critical pathological, physiological, and biochemical insights, alongside a valuable number of specimens for further study [49,50].

A breakthrough was achieved by the Michael Houghton team, which constructed a lambda phage library from cDNA of a high-titer chimpanzee plasma specimen [51]. The Michael Houghton team screened more than 1 million expression clones with serum from a chronic NANBH patient, which led to identification of a single positive cDNA clone, designated 5-1-1, marking the discovery of HCV [51]. This led to the development of initial assays for detecting anti-HCV antibodies, incorporating the 5-1-1 antigen [52]. As a result of this seminal work, the 2020 Nobel Prize in Medicine or Physiology was awarded to Harvey Alter, Michael Houghton, and Charles Rice for their seminal work in the discovery and characterization of HCV [53].

Characterization of the newly discovered virus revealed that the HCV genome comprises a positive-strand RNA of nearly 10,000 nucleotides, with a single open reading frame (ORF), closely related to the *Flaviviridae* family [54,55]. Identification of the 5′ and 3′ untranslated regions (UTRs) facilitated the production of a full-length cDNA clone, infectious upon intra-hepatic administration in chimpanzees [56].

Despite advancements in screening and the prevention of hepatitis C, it remains a significant global health concern [1,31]. Continuous investigations involving HCV life cycle, replication, mechanisms of persistence, and pathogenesis are still needed to unravel the complexity of this infection, which would be helpful in the eradication efforts [57]. The recent estimates point to slightly less than 1% of the global population as chronically infected with HCV, and while treatment efficacy and accessibility have tremendously improved, HCV infection continues to be a leading cause of morbidity and mortality worldwide [58].

## 3. HCV Origin and Classification

HCV is a member of the *Hepacivirus* genus within the *Flaviviridae* family, a classification delineated through seminal studies led by Peter Simmonds [59,60,61,62]. A notable review conducted by Peter Simmonds [63] meticulously examined the complex narrative surrounding the origins and evolutionary trajectory of HCV. The aforementioned review highlighted the challenges to pinpoint the ancient history of HCV in human populations and recommended a cautious interpretation of the term “origin”, particularly in reference to HCV global dissemination and diversification during the 20th century [63]. The absence of an animal virus closely related to HCV, despite exhaustive efforts, further complicates delineating the exact zoonotic source of HCV in humans [62,63,64].

**Figure 1:**
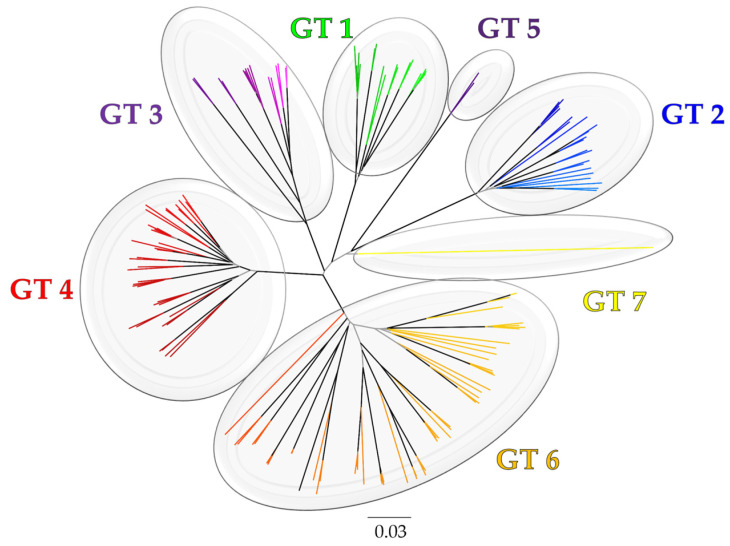
The evolutionary history of HCV genotypes (GTs) based on (Sallam, 2017) [66]. The phylogeny was constructed using the neighbor-joining (NJ) method with bootstrap test for evaluation of topology (1000 replicates). Internal branches with bootstrap values ≥ 0.9 are highlighted in black. The evolutionary distances were computed using the TN93 method. The rate variation among sites was modelled with a gamma distribution (shape parameter = 4). The analysis involved 147 *NS5B* sequences (1495 bases) downloaded from the Los Alamos Hepatitis C sequence and immunology databases (https://hcv.lanl.gov/content/index, accessed on 30 April 2024). Evolutionary analyses were conducted in MEGA6 [68].

In exploring the genetic heterogeneity of HCV, phylogenetic studies revealed a remarkable divergence within the virus, categorizing HCV into at least seven (or even eight) distinct genotypes, denoted by Arabic numerals [60,65,66,67] (Figure 1).

This genetic diversity extends within genotypes, which resulted in further subclassification of these genotypes into several subtypes identified by lowercase English letters [60,65]. These genotype and subtype classifications are based on variations in nucleotide sequences, with inter-genotypic differences exceeding 30% and intra-genotypic differences ranging between 20 and 25% [24,60,65]. Investigation of the evolutionary rate of HCV revealed a relatively rapid rate, estimated at 1.0–2.0 × 10^−3^ substitutions per site per year (s/s/y), comparable to other RNA viruses [69]. Given the extensive genetic divergence of HCV isolates, it is plausible that such variability underpins the observed differences in clinical manifestations and treatment responses among individuals infected with distinct HCV genotypes [70,71,72,73].

## 4. HCV Genome

The genomic architecture of HCV exemplifies a *Flaviviridae* member in being a positive-sense, single-stranded RNA genome, constituting a single ORF [74]. This ORF is responsible for the synthesis of a large single polyprotein, approximately 3000 amino acids in length, as delineated in the work by Li et al. and Scheel and Rice [75,76]. The processing of HCV polyprotein into its functional single proteins involves a series of cleavage events mediated by both host cellular proteases and virus-specific enzymes, yielding an array of structural and non-structural (NS) proteins critical for the viral life cycle [76,77].

The details of the HCV genomic regions can be further explained as follows. Positioned at the beginning of the HCV viral genome is the highly conserved 5′ UTR, which harbors an internal ribosomal entry site (IRES) facilitating the cap-independent translation of the viral genome [78]. This region precedes the coding sequences for structural proteins, including the core protein (C), the envelope glycoproteins (E1 and E2), and the ion-channel viroporin (p7) [78]. Notably, the C protein maintains a conserved sequence, whereas E1 and E2 glycoproteins exhibit significant sequence variability, a phenomenon attributed to the pressure of immune selection [70,79,80,81].

**Figure 2:**
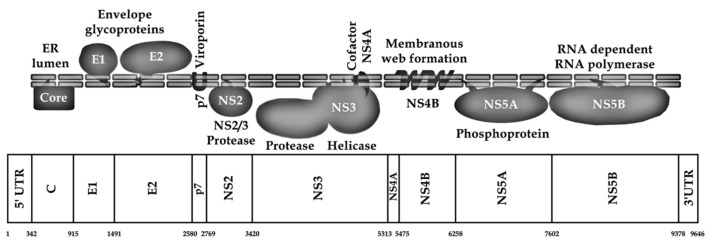
Schematic illustration of HCV genome and polyprotein. The nucleotide positions are in accordance with strain H77 numbering (GenBank accession number: AF009606) based on (Sallam, 2017) [66].

The HCV genome’s last segment encodes NS proteins, essential for viral replication and processing of the polyprotein [82]. The NS2 protein, a cysteine protease, facilitates the cleavage of NS3 from the NS2–NS3 junction [83]. NS3, together with NS4A, constitutes a serine protease complex essential for the cleavage of subsequent NS proteins, regulating the sequential processing of the viral proteome [76]. NS4B serves as a membrane anchor for the viral replication complex, while NS5A, alongside NS4B, contributes to the formation of the endoplasmic reticulum (ER) membranous web, a structure essential for viral replication dynamics [76,84]. Lastly, NS5B acts as an RNA-dependent RNA polymerase (RdRp), synthesizing the viral RNA genome and acting as the central replication factor of HCV [85,86]. Full representation of the HCV genome and viral proteins is illustrated in (Figure 2).

## 5. HCV Pathogenesis, Pathology, and Immune Response

The hepatic tropism of HCV is related to its selective predilection to infect the hepatocytes as the primary site for its entry, replication, and assembly [87]. The primary mode of HCV transmission is identified as percutaneous, with documented cases of permucosal transmission as well [88]. Experiments showed that direct inoculation of HCV, either through intravenous injection of virions or intrahepatic introduction of genomic RNA, effectively initiates HCV infection [56].

Hepatic tropism of HCV is related to the preferential expression of specific proteins on hepatocytes acting to facilitate entry and replication of HCV. These include the low-density lipoprotein receptor (LDL-R) and scavenger receptor class B type I (SR-BI), the liver-specific microRNA miR-122 essential for HCV replication, and the unique lipoprotein synthesis pathways in the liver that support HCV virion production [89,90,91]. Additionally, the liver-specific distribution and dynamic interactions between viral components and more universally expressed host proteins including CD81, claudins, occludin, and epidermal growth factor receptor further highlight the role of the liver as the principal site for the HCV life cycle [92,93]. While the potential for productive HCV infection in extrahepatic cells remains under investigation for full elucidation, there is evidence supporting the detection of HCV replicative intermediates outside the liver, suggesting a minor yet evident extrahepatic replication compartment [94,95,96,97].

Following HCV infection, the host defense mechanisms, including the innate and adaptive immune responses, restrict the penetration of circulating HCV particles into hepatocytes [87,98]. HCV demonstrates an ability to adapt and evolve in response to the immunological challenges imposed by the host environment [99]. To circumvent the humoral responses, intrahepatic cell-to-cell transmission is utilized by the virus [100]. This complex dynamic interaction between HCV evasion strategies and the host immune response is the key feature of the outcome following HCV infection [101,102]. Understanding the mechanisms behind HCV intra-liver dissemination and its interaction with host immune defenses is crucial for developing targeted therapeutic strategies aimed at controlling viral spread and enhancing the efficacy of antiviral treatments [98].

The innate immune response plays a crucial role in fighting HCV infection, with the virus employing various strategies to circumvent this defense [100]. Central to the innate immune response against HCV is interferon (IFN) signaling, which constitutes early intrahepatic defenses [103]. Activation of IFN-regulatory factor 3 (IRF3) by viral infection triggers IFN-β production, subsequently activating the JAK-STAT signaling pathway, enhancing IFN-α synthesis, and stimulating the release of antiviral cytokines and chemokines [104]. The HCV NS3–NS4A complex disrupts IRF3 activation by cleaving key adapter protein Toll/IL-1 receptor domain-containing adaptor inducing IFN- β (TRIF), integral to the Toll-like receptor 3 signaling pathway [105]. This interference highlights the HCV ability to evade antiviral immunity, a process counteracted by NS3–NS4A protease inhibitors [106].

Additionally, the NS5A protein can disrupt IFN signaling through various mechanisms, including the stimulation of interleukin 8 (IL-8) production and inhibition of the antiviral proteins [107]. The involvement of natural killer (NK) and NKT cells, which are abundant in the liver, is significant for HCV control, with their activity potentially inhibited by HCV E2 protein interaction with CD81 and influenced by HLA and killer immunoglobulin-like receptor (KIR) interactions [108,109]. Moreover, robust CD4 and CD8 T-cell responses are essential for HCV control, although their failure to prevent chronicity of HCV infection remains poorly understood and needs further investigation [110]. HCV-specific T cells emerge swiftly during acute infection and can persist in individuals following HCV infection clearance [111]. In chronic infection, stronger CD8 T-cell responses correlate with reduced hepatitis C viral loads [112]. 

Spontaneous clearance of HCV has been correlated with specific major histocompatibility complex (MHC) class I and class II molecules [98]. Studies identified a positive association in terms of higher probability of virus clearance with certain alleles, including HLA-B27, HLA-B57, HLA-A11, HLA-A03, and HLA-Cw01, and a negative correlation in terms of higher possibility of HCV persistence with HLA-B18 and HLA-Cw04 [113,114,115,116,117,118]. Nevertheless, these associations may vary depending on the circulating HCV genotypes [119]. Although the exact mechanisms through which these alleles can influence HCV clearance remain largely undetermined, it is proposed that protective alleles may effectively present immunogenic and conserved epitopes to T cells, enhancing viral clearance [120]. Conversely, alleles associated with an increased risk of chronic infection might act as ligands for inhibitory receptors on NK cells, with supporting evidence suggesting a significant role for NK receptor polymorphisms in HCV clearance [108].

The correlation between specific MHC class II genes and HCV clearance adds further emphasis to the role of host genetics in the outcome of HCV infection [121,122]. Research indicates that individuals who end up clearing the HCV infection tend to target a broader range of MHC class II epitopes compared to individuals who develop chronic HCV infection, targeting a smaller number of epitopes, which suggests a potential discrepancy in antigen processing or epitope presentation [123].

Most acute HCV infections (55–85%) become chronic due to the virus’s effective evasion strategies, with spontaneous clearance being rare once chronicity is established [4,124,125]. Specifically, a large study by Bulteel et al. reported that spontaneous clearance of HCV occurred at an incidence rate of 0.36/100 person-years follow-up [125]. Thus, the majority of HCV-infected individuals progress to chronic infection despite robust T-cell responses [111]. The HCV persistence is facilitated by selection for mutations that evade immune responses while maintaining replicative fitness [126,127]. These mutations often occur in cytotoxic T-lymphocyte (CTL) epitopes and are associated with impaired MHC class I presentation or reduced TCR contact [128].

The humoral immune response also plays a critical role in controlling HCV infections, with neutralizing antibodies primarily targeting the E2 protein [129]. These antibodies can influence the viral fitness and sequence evolution during acute infection, suggesting their significant impact on viral dynamics [130]. Despite the high variability of HCV and mechanisms like glycan shielding and lipid hiding that aid in immune evasion, neutralizing antibodies decrease infection risk post-exposure and modulate disease progression [131,132,133].

The role of virus factors reflected in the extreme genetic diversity of HCV does not appear to significantly alter the clinical manifestations across different HCV genotypes; nevertheless, genotype-specific responses to different treatments and associations with conditions such as steatosis vary among different genotypes [24,134,135].

## 6. Epidemiology, Transmission, and Global Figures of Hepatitis C

HCV primarily leads to severe health complications such as liver failure and HCC, particularly through its progression to chronic infection [136]. The risk of these severely morbid conditions increases with age and the length of time a person has been infected with the virus [137,138]. The WHO reported that in 2022, around 242,000 deaths were attributed to hepatitis C, which highlights the significant burden of the disease [4].

The spread of HCV infection during the 20th century was significantly influenced by the increased production and global use of syringes, initially for medical purposes and subsequently for illicit drug use among injection drug users (IDUs) [139,140]. Before the recognition of blood-borne transmission, the widespread adoption of percutaneous injections played a crucial role in disseminating HCV globally, notably elevating infection rates by 5 to 20 times in regions with prevalent unsafe injection practices, particularly among IDUs [141,142]. Additionally, the transfusion of unscreened blood products further facilitated the spread of HCV before virus identification [143]. Thus, the widespread transmission of HCV throughout the 20th century was largely fueled by the increased production and use of syringes for both medical purposes and illicit drug use, highlighting the impact of healthcare practices on the spread of bloodborne pathogens [141]. 

Historically, before the 20th century, practices such as scarification rituals and circumcision likely maintained HCV transmission [144]. This hypothesis is supported by current evidence of HCV transmission through these practices in certain world regions (e.g., in Egypt) and molecular clock analyses of HCV RNA sequences [145,146].

Transmission of HCV occurs mainly through percutaneous exposure to infected blood [147]. HCV can also spread from mother to infant (vertical transmission) and less frequently via sexual contact, especially among men-who-have-sex-with-men (MSM) [148,149,150]. There is no risk of HCV transmission through intact skin; however, transmission can happen if infected blood contacts mucosal surfaces, such as the eyes [151].

The probability of HCV transmission is directly related to the volume of blood involved in transmission and the nature of exposure to the virus inoculum [152]. In acute or chronically infected individuals, the blood usually harbors 3 to 6 log copies of HCV RNA per mL [153,154]. Thus, the blood constitutes the main route for HCV transmission, with a lower likelihood of transmission if the blood is not viremic [155]. While HCV RNA can be detected in other body fluids, the potential of these fluids to transmit HCV infection remains unclear [156,157].

High-risk HCV transmission scenarios often involve substantial percutaneous exposures, such as through blood transfusions of contaminated blood [140,147]. Nevertheless, even small volumes of blood can transmit HCV if introduced percutaneously, especially in healthcare settings where strict infection control practices may not be adhered to consistently [158,159,160,161]. Overall, the per-act risk of HCV transmission through needlestick injuries has been reported at a rate of 1% to 2% [162,163]. Repeated, small-volume exposures to contaminated blood explain the high incidence of HCV among IDUs [164]. Non-medical percutaneous exposures, such as tattooing, body piercing, and wet cupping (hijama), are also associated with HCV transmission and are more prevalent in certain countries, though these factors can be confounded by other high-risk behaviors [165,166].

The role of sexual transmission in HCV is less conspicuous, as it appears to be rare among long-term monogamous couples with higher occurrence among MSM [167,168]. Transmission from an infected mother to her infant is less common, occurring in about 4% to 8% of cases [169].

**Figure 3:**
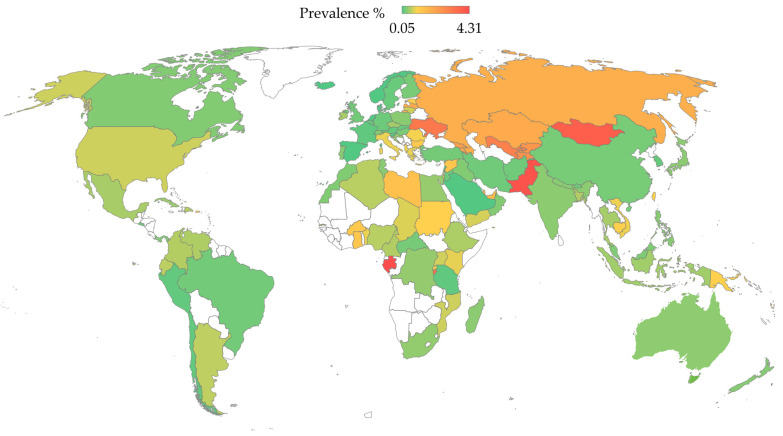
The prevalence of HCV per country based on the Polaris Observatory data [174]. The map was generated in Microsoft Excel, powered by Bing, © Australian Bureau of Statistics, GeoNames, Microsoft, Navinfo, Open Places, OpenStreetMapTomTom, Zenrin. The map of Australia was generated by an older version of Microsoft Excel, powered by Bing, © GeoNames, Microsoft, Navinfo, TomTom, Wikipedia. We are neutral with regard to jurisdictional claims in this map.

Hepatitis C is a major global health challenge, being one of the primary causes of liver disease [170,171]. The WHO’s latest estimates report that there are approximately 50 million people infected with HCV globally, with around 1 million new infections each year and about 242,000 deaths annually attributed to HCV-related complications [4]. The prevalence of HCV varies significantly across different regions and among various age and risk groups [172,173]. In 2022, the highest number of total HCV infections was reported in Pakistan, followed by India, China, Russia, and the United States, while the prevalence was >3.0% in Gabon, Pakistan, Mongolia, Burundi, Ukraine, and Uzbekistan (Table 1 and Figure 3) [174].

Historically, Egypt has experienced the highest prevalence of HCV, particularly among individuals born before 1960, where rates among this particular age demographic had reached as high as 50% [175]. This high prevalence can be traced back to public health campaigns from the 1950s to the 1980s aimed at eradicating schistosomiasis [176]. These campaigns involved the mass intravenous administration of tartar emetic using equipment that was often reused and inadequately sterilized, leading to widespread HCV transmission [177].

Currently, China, India, and the United States represent countries with a high burden of HCV disease globally [2]. These three countries lead in the number of HCV cases, surpassing Egypt, which was among the top until 2019 [178,179].

## 7. Genetic Diversity of HCV

Shortly after the discovery of HCV, it became evident that distinct genetic strains of the virus existed in different geographical areas [180,181,182]. The HCV nomenclature standards—which were pioneered by the prominent virologist Peter Simmonds—have identified seven major genotypes of HCV, which are phylogenetically distinct, with further divisions into subtypes within these genotypes [60,65,183]. The HCV genotypes and subtypes display variations in their response to treatments. For example, genotypes 1 and 4 show less responsiveness to IFNs, while the lower susceptibility of genotype 3 to DAAs remains a challenging issue [184,185,186]. Additionally, HCV genotypes demonstrate variability in clinical outcomes beyond their influence on antiviral drug resistance. For example, genotype 3 is notably associated with an increased occurrence of liver steatosis, which involves significant fat accumulation within the liver as well as an increased progression to fibrosis and cirrhosis [187,188,189,190]. On the other hand, subtype 1b has been linked with a higher risk of HCC development [191,192].

The HCV genomic nucleotide difference among genotypes is higher than 30%, with approximately 15% genetic difference among subtypes [24,65]. It is important to point out that these genetic differences are not evenly distributed across the HCV genome since these differences are most pronounced in the E1, E2, and the V3 region of NS5A proteins, whereas the C protein shows less variability [24,193].

Despite the possible occurrence of recombination in HCV, inter-genotypic recombination is reported rarely; therefore, reconstruction of HCV phylogeny is considered reliable for the majority of sub-genomic HCV regions reflecting the clustering patterns of full genome analysis [194].

For the HCV genetic diversity at the global level, a notable variation has been reported [173]. In spite of this variability, genotype 1 is common globally, with phylodynamic studies showing that the global dissemination of this genotype took place between the 1940s and 1980s [195]. Evidence also suggested that subtype 1b may have spread earlier than subtype 1a [195]. Subtype 1a is frequently associated with IDUs, specifically in North America and Northern Europe, while subtype 1b is often associated with a history of blood product use [196,197].

Local epidemics of HCV often exhibit dominance of specific subtypes, largely due to founder effects rather than variations in how easily the virus is transmitted or the routes of transmission [198,199]. For example, the HCV epidemic in Egypt is predominantly driven by subtype 4a [200]. In contrast, West Africa demonstrates significant diversity within genotype 2, indicating it likely originated there [201]. In Southeast Asia, genotypes 3 and 6 predominate, especially among IDUs [202,203,204,205]. Meanwhile, genotype 5 is most frequently found in Southern Africa [206,207,208].

Regarding the origin of HCV in humans, recent discoveries of hepaciviruses in non-primates like dogs and horses show that these are distinctly different from HCV [209,210,211,212]. This suggests a complex and ancient evolution of HCV in humans, with origins that potentially date back hundreds or even thousands of years [63,145]. However, without older sequence data, the precise dating of HCV emergence remains challenging due to calibration uncertainties in molecular clock analyses [213]. 

## 8. Clinical and Histopathologic Features of Hepatitis C

Acute HCV infection often proceeds sub-clinically, with a significant proportion of cases remaining asymptomatic [214]. However, the onset of acute hepatitis C can manifest in a subset of infected individuals, with symptoms characteristic of acute viral hepatitis, including malaise, fatigue, anorexia, nausea, abdominal pain, jaundice, dark urine, and pale stools [215]. Notably, fulminant hepatitis, a severe and rapid deterioration of liver function, appears to occur rarely in the context of HCV [216,217].

The interval from HCV exposure to the appearance of symptoms or laboratory evidence of liver injury (incubation period) can vary widely, ranging from two to twenty weeks and typically manifest in seven weeks [218,219]. Frequently, the primary laboratory indication of acute HCV infection is elevated levels of alanine aminotransferase (ALT) and aspartate aminotransferase (AST), indicative of damaged hepatocytes [220].

Following HCV infection, viremia is rapidly detectable, rising to between 100,000 and 10 million IU/mL within a few weeks [153,221,222]. A decline in viral load often follows, occurring one to two weeks later and coinciding with a marked increase in ALT and AST levels [153,223]. This pattern reflects the immune system response to HCV infection, which mediates hepatocyte destruction and, importantly, influences the rate of spontaneous viral clearance [224,225]. Consequently, higher initial HCV viremic levels and more severe forms of acute hepatitis C are correlated with higher likelihoods of spontaneous clearance [226].

Spontaneous clearance of HCV typically manifests within six months of infection [227]. A higher probability of viral clearance has been observed in symptomatic individuals, females, and younger patients [228,229,230,231,232]. Conversely, spontaneous resolution of HCV infection is less likely to be observed among those co-infected with HIV, IDUs, or individuals of black ethnicity [233,234]. The identification of specific alleles near the *IL28B* gene, responsible for encoding interferon-lambda 3 (IFNλ3), has provided important insights into the genetic determinants of HCV infection outcomes [235,236,237]. These alleles are also predictive of spontaneous recovery from acute HCV [238,239]. Significantly, the distribution of this protective *IL28B* genotype (CC genotype as opposed to the CT/TT genotypes) exhibits marked racial variations, being more prevalent in Asian populations and notably less so in those of African descent [240].

In the vast majority of individuals (60% to 85%) who do not undergo spontaneous HCV infection resolution, chronic infection emerges as a highly variable condition, characterized by diverse clinical manifestations and progression rates [227,241]. Significant morbidity and mortality typically arise when the infection advances to cirrhosis or end-stage liver disease, which may further evolve into HCC [242,243,244]. 

Among chronically infected HCV patients, the levels of HCV RNA in the blood are relatively constant over time, generally fluctuating, and can reach over 1 million IU/mL [245]. Certain factors are associated with higher viral loads, including HIV co-infection, male gender, older age, and increased body mass index [246,247]. Conversely, individuals with concurrent HBV infection or more advanced liver disease tend to exhibit lower viral loads [248].

Chronic HCV infection induces a spectrum of histopathological changes in the liver, characterized primarily by variable levels of chronic inflammation and steatosis [249]. Frequent periportal lymphocytic infiltrates are noted but do not consistently predict the course of liver disease progression [249]. In chronic hepatitis C, a disparity in the development of significant fibrosis is noted where some individuals exhibit marked fibrotic changes after prolonged viral exposure, while others show minimal effects, the reason of which remains to be fully elucidated [247,250,251].

Fibrosis develops as a result of an imbalance in extracellular matrix dynamics, with collagen production exceeding its breakdown, initiating in the periportal zones [252]. This fibrosis process may stabilize or advance to more severe structural alterations, including the formation of septae that bridge adjacent lobules [253]. Progression beyond this stage culminates in cirrhosis, characterized by extensive scarring and nodular liver regeneration [254].

As cirrhosis advances, complications such as portal hypertension develop, and there is an escalated risk of HCC due to neoplastic transformations within the hepatic parenchyma [255,256]. Understanding this pathophysiological process that culminates in end-stage liver disease is necessary for timely and effective therapeutic interventions to halt or reverse the fibrosis processes in chronic HCV infection [257,258].

The potential for HCC in chronic HCV infection constitutes a significant risk, necessitating vigilant screening protocols for cirrhosis and HCC [259]. This proactive approach via HCV screening is crucial due to the typically asymptomatic progression of chronic HCV until the patients reach advanced stages [260].

The role of HCV in the direct induction of oncogenic processes remains under investigation [261,262,263]. There is growing evidence that the HCV C protein engages in oncogenic modulation by activating proto-oncogenes and suppressing apoptotic pathways [264,265]. However, it is also plausible that the chronic inflammation, which is the hallmark of persistent HCV infection, sufficiently promotes oncogenesis, leading to the development of HCC [266,267].

Finally, chronic HCV infection is implicated in a variety of extrahepatic manifestations predominantly inflammatory in nature [96,268]. These manifestations further complicate the clinical management of HCV, illustrating the need to address the broader systemic effects of chronic HCV infection [269]. 

## 9. Diagnosis of HCV

Infection by HCV should be considered in patients presenting with unexplained liver abnormalities [270]. Given the relatively common prevalence of chronic hepatitis C and the frequently asymptomatic nature of the disease, HCV testing is warranted in individuals displaying elevated liver transaminases or those with risk factors for HCV acquisition [271]. Importantly, the shared nature of risk factors between HCV and HIV necessitates vigilant screening protocols for this particular group, namely HIV-infected individuals [272]. Accordingly, HCV screening for all individuals diagnosed with HIV is advocated as a strategy aimed at early detection and management of HCV to prevent further liver damage and optimize patient outcomes [5].

The recent guidelines from the IDSA, the AASLD, and the U.S. Centers for Disease Control and Prevention (CDC) endorsed universal HCV screening in response to evolving epidemiological trends and the availability of effective treatments [13]. As of March 2020, it is recommended that all adults between the ages of 18 to 79 undergo HCV screening [5]. This policy was further expanded in April 2020 by the CDC to include a one-time screening for all adults aged 18 and older and for every pregnancy, barring settings where the HCV prevalence is below 0.1% [271].

The adoption of universal HCV screening represents a strategic evolution in public health policy, driven by a variety of factors [273]. The evident cost-effectiveness of widespread screening initiatives has established them as practical and essential public health interventions [274,275]. Additionally, universal screening significantly enhances the detection of HCV, which is particularly crucial given the often-silent progression of the infection [276]. The urgency for a universal screening protocol is emphasized by the increasing incidence of HCV, especially among younger populations, where the disease may otherwise go unnoticed until advanced stages [277,278]. The current availability of safe and cost-effective DAAs ensures that HCV can be treated efficiently following detection [279]. Effective treatment also contributes to halting the forward transmission of the virus, further reducing the HCV burden through TasP [280].

The initial diagnostic approach for HCV infection involves serologic testing through Enzyme Immunoassays (EIAs), which detects antibodies against HCV C, NS3, NS4, and NS5 proteins [281]. Despite its high specificity and sensitivity, EIAs’ results can occasionally yield false positives [281,282]. Therefore, positive serologic results necessitate further confirmation through HCV RNA testing to determine active infection [283,284].

Serological testing for HCV is diverse, employing a range of technologies from EIA and chemiluminescence immunoassay to rapid techniques such as agglutination and lateral flow assays [281,285]. Advanced methodologies include recombinant immunoblot assay (RIBA), electrochemical immunosensors, and nano-metal technologies utilizing gold nanoparticles and quantum dots [286,287,288]. Despite the technological advances in HCV screening, several challenges hinder its widespread application, particularly in resource-limited environments [281]. These issues include the prolonged turnaround times and the substantial costs associated with these tests, which are compounded by the bulky nature of the instrumentation and the necessity for skilled technicians [289]. Such constraints have motivated innovation in diagnostic approaches that balance accuracy and practicality, aiming to reduce both equipment costs and operational complexity while maintaining test sensitivity and specificity [290]. 

HCV RNA testing remains crucial for cases with elevated clinical suspicion, facilitated by methods like reverse transcriptase-polymerase chain reaction (RT-PCR), transcription-mediated amplification (TMA), reverse transcription loop-mediated isothermal amplification (RT-LAMP), and branched DNA (bDNA) assays [291,292,293,294]. These methods, targeting highly conserved regions of the HCV genome, are critical for confirming the chronic infection status and informing treatment strategies.

Genotyping of HCV was crucial for tailoring IFN-based therapy, and this involved utilization of reverse hybridization assays that amplify specific genomic regions (*5′UTR* or *C*) to determine genotype and occasionally subtype [281,295]. However, the most reliable method for HCV genotype determination employs genomic sequencing and phylogenetic analysis of the *E1* or *NS5B* regions [296]. The role of HCV genotyping following the availability of pan-genotypic DAA regimens remains an issue to be further investigated [135].

Accurate staging of HCV-related liver disease, which historically relied on invasive liver biopsies, is essential [297]. Despite the detailed insights of liver biopsies, this invasive approach carries risks and limitations [298]. The shift towards non-invasive methods like serum markers and sonographic elastography is reshaping HCV management [299,300,301,302]. This allows more frequent and less invasive monitoring, providing a clearer assessment of liver fibrosis without the drawbacks associated with traditional biopsy methods [303]. 

## 10. Treatment of HCV

The therapeutic approach for HCV infection experienced a significant transformation, particularly with the advent of DAA, with a notable shift from IFN-based treatments to more effective and tolerable options with shorter durations of treatment [22]. Historically, the standard-of-care for HCV management involved pegylated IFN and ribavirin [304]. This option was often effective in achieving an SVR; nevertheless, it was complicated by its lengthy duration and often led to considerable side effects [305].

The introduction of DAAs marked a revolutionary step in HCV management, with cure rates above 95% and defining SVR, specifically undetectable HCV RNA 12 weeks post-treatment (SVR12), as a new clinical benchmark instead of 24 weeks [306,307]. Additionally, 8-week regimens have shown effectiveness in real-world studies, with a positive impact on treatment adherence and reduction in costs [308,309,310]. DAAs offer the enhancement of patient tolerability to treatment and allow for the customization of treatment regimens based on individual patient factors such as HCV genotype, stage of liver fibrosis, co-existing medical conditions, prior treatment history, and potential RASs [311,312]. Despite the clinical success of DAAs, a limitation is related to their high cost which is a critical consideration, especially in low- and middle-income countries [21].

Historically, the efficacy of IFN-based treatments was influenced by a range of factors, including patient demographics, viral characteristics, and genetic markers such as *IL28B* polymorphisms [304]. Additionally, IFN and ribavirin therapies were well-known for their broad and severe side effect profiles [313]. Common IFN adverse effects reported in large trials included fatigue, headache, nausea, insomnia, and pyrexia, along with more severe impacts such as anemia, neutropenia, and a range of psychiatric and immunological reactions [314]. Notably, ribavirin frequently caused hemolytic anemia, a challenging complication that often necessitated dose adjustments or discontinuation of therapy [315].

These side effects underline the challenges of the older treatment regimens and highlight the advantages of DAAs, which have fewer adverse effects and do not require the intensive monitoring and management that IFN-based therapies did [316]. Thus, the introduction of DAAs revolutionized the treatment of chronic HCV infection, marking a significant milestone in the management and possible eradication of the disease as a public health threat. The U.S. Food and Drug Administration (FDA) approved the first DAAs, telaprevir and boceprevir, in May 2011 [317]. Approval of other DAAs followed, which represented a major therapeutic breakthrough, marking a new era of hepatitis C treatment characterized by enhanced efficacy, tolerability, and shorter duration [318]. A timeline of DAAs’ development and approval is presented below:

2011: Introduction of boceprevir (Victrelis) and telaprevir (Incivek), pioneering the DAA classes with enhanced direct antiviral activity against HCV [319].

2013: Approval of sofosbuvir (Sovaldi), a landmark in DAA therapy characterized by high cure rates, reduced side effects, and shorter treatment durations [320].

2014: The FDA approved combination therapies such as ledipasvir/sofosbuvir (Harvoni), simplifying HCV treatment by eliminating the need for interferon and shortening the course of therapy [321].

2016: Introduction of pan-genotypic treatments such as elbasvir/grazoprevir (Zepatier) and sofosbuvir/velpatasvir (Epclusa), capable of treating all HCV genotypes effectively [322].

2017: Approval of glecaprevir/pibrentasvir (Mavyret), enhancing the treatment with shorter courses to achieve SVR and pan-genotypic high efficacy [323].

Thus, the introduction of DAA agents was a substantial progression from the previous treatment modalities [324]. The DAAs directly target specific steps within the HCV replication cycle. Clinical evidence robustly supports the efficacy of DAA regimens, with data indicating that over 95% of patients treated with DAAs achieve an SVR, which is clinically equated with virological cure [325,326,327,328,329,330]. A summary of four common, currently approved DAA regimens is presented in (Table 2).

Innovative therapeutic strategies are being explored as well, to expand the treatment options for HCV especially for challenging cases (e.g., patients with HCC) as reviewed comprehensively by Medina et al. [343]. For example, agents such as ezetimibe that target cellular cholesterol, which is critical for viral entry, can offer a novel mechanism to prevent HCV entry into hepatocytes [344]. Moreover, clinical trials incorporating statins with DAAs or IFN are underway [345]. These trials aim to enhance the antiviral response by taking advantage of the effects of statins on lipid metabolism, which may also boost IFN effectiveness [343].

## 11. Prevention of HCV

Infection with HCV remains a challenging public health threat [57]. The burden of HCV necessitates various strategies for prevention [1]. Key preventive strategies to reduce the burden of HCV include improved screening, harm reduction in the context of IDU, prevention of healthcare-associated infections, and the use of TasP [346,347,348,349,350].

Universal HCV screening with concomitant treatment of the detected cases is recommended, considering the natural history of chronic hepatitis C where the disease is often asymptomatic until advanced liver disease develops [13]. Recommendations advocate for one-time screening for all individuals aged 18 years and routine screening for high-risk populations, including individuals with an IDU history and individuals on long-term hemodialysis [13,351]. Early diagnosis of HCV infection through widespread screening facilitates timely access to treatment [352]. Additionally, there is growing evidence supporting the cost-effectiveness of universal HCV screening even in countries with low HCV prevalence [353,354].

Regarding harm reduction, needle exchange programs (NEPs), opioid substitution therapy (OST), and educational programs have shown potential to reduce HCV transmission—in addition to other bloodborne viruses—among IDUs [355,356]. In healthcare settings, strict adherence to proper infection control practices is important to prevent HCV transmission, including proper sterilization of medical and dental equipment [357]. Importantly, the implementation of TasP appears crucial to reduce the burden of HCV infections [350]. Besides the dramatic improvement in the treatment of chronic HCV, the DAAs stimulated efforts towards its global elimination [358]. The absence of an effective HCV vaccine has led to the adoption of the TasP strategy [359]. TasP advocates for the widespread and prompt treatment of HCV infections to substantially lower HCV transmission within populations, thereby reducing the prevalence of hepatitis C [360].

However, the effective implementation of TasP faces significant challenges, particularly regarding the development of drug resistance [361]. RASs can occur naturally or develop during treatment, presenting a challenging obstacle and reducing treatment efficacy [17]. The presence of RASs may limit the scalability of treatment programs since therapies proven effective in clinical trials may exhibit diminished effectiveness in real-world applications due to these resistant strains [362].

Additionally, the spread of drug-resistant variants is particularly concerning in high-risk groups, such as IDUs, who are more prone to disseminating these variants [363,364]. Thus, there is a need for continuous surveillance and the development of novel therapeutic options that can bypass HCV resistance mechanisms [21].

## 12. Resistance-Associated Substitutions (RASs)

As mentioned earlier, the therapeutic strategy for chronic HCV infection has been revolutionized by the approval of DAAs, which present dramatically improved cure rates over IFN-based therapies across various HCV genotypes [365]. Nevertheless, the success story of DAAs as a curative therapy for HCV can be undermined by RASs conferring resistance [362]. This feared outcome is related to the high mutation rate inherent to HCV due to its error-prone replicase enzyme (RdRp) [366,367]. The RASs could arise from mutations occurring spontaneously due to the natural genetic diversity of the virus (natural resistance) or be induced under the selective pressure exerted by antiviral therapies (acquired resistance) [368,369,370]. Besides the issue of emerging RASs to DAAs, other factors contribute to treatment failure, including patient adherence to treatment and suboptimal treatment regimens [36].

The prevalence of RASs is influenced by both viral genotype and geographic factors [35,371]. In an early comprehensive analysis utilizing published GenBank data, the global prevalence of resistance-associated variants (RAVs) to DAAs was determined [372]. The study revealed that a significant proportion, 58.7% (854 out of 1455 sequences), harbored at least one dominant resistance variant, with notable geographic discrepancies [372]. Asia exhibited the highest frequency of RAVs at 74.1%, followed by Africa at 71.9%, America at 53.5%, and Europe at 51.4% [372]. Among the HCV genotypes, genotype 6 displayed the highest frequency of RAVs at 99%, a notably high prevalence compared to other genotypes. This was followed by genotype 2 at 87.9%, genotype 4 at 85.5%, subtype 1a at 56%, genotype 3 at 50%, and subtype 1b at 34.3%. The study also assessed the distribution of RAVs across different classes of DAAs. It was found that 40.0% of sequences contained RAVs associated with NS5A inhibitors, and 29.6% with NS3 inhibitors, highlighting a significant challenge in managing resistance to these therapies [372]. In contrast, resistance to NS5B NIs and NI-based combinations was notably lower, with less than 4% of sequences showing RAVs [372].

Defining the clinical significance of RASs remains a significant challenge [373]. For example, RASs identified in phenotypic assays during cell culture studies with selective pressure by DAAs do not always correspond to those emerging in clinical scenarios where failed treatment is manifested [373]. Thus, not all RASs detected through sequencing directly impact the efficacy of DAA therapies [374]. Furthermore, the significance of RAVs within the HCV quasispecies is a critical factor in understanding resistance dynamics [375]. Variants below a 15% frequency within the HCV quasispecies generally exert minimal influence on treatment outcomes [32]. This observation is essential for clinical practice, as it informs the threshold of variant detection that should concern clinicians, guiding more relevant approaches to the use of DAAs [373]. 

## 13. HCV Vaccination Challenges

Despite the revolutionary impact of DAAs on HCV treatment, the quest for a preventive hepatitis C vaccine continues to be a crucial public health issue [376]. While DAAs have remarkable effectiveness, these drugs carry a high cost burden and face distribution challenges that limit their accessibility, especially in low-income settings [377,378]. Furthermore, the curative treatments do not confer immunity against future infections, an issue of particular concern in populations engaged in high-risk behaviors (e.g., IDUs) [379]. A vaccine would dramatically reduce the incidence of new infections and hinder HCV transmission within communities, offering a sustainable and cost-effective strategy to mitigate the global HCV epidemic [380].

However, the development of an effective HCV vaccine is hampered by the high genetic diversity of HCV, which enables the effective escape from immune recognition [381,382]. This diversity complicates the identification of universal vaccine targets and restricts vaccine epitope design [383,384].

Promising vaccine strategies like the use of cyclic peptides, which hold potential for eliciting strong neutralizing antibody responses, face hurdles in development of the delivery systems necessary to maximize their immunogenicity [385]. Additionally, the absence of robust infection models that accurately mimic human HCV infection presents a significant barrier to assessing vaccine efficacy [23,386].

Recent advances in HCV vaccine research yielded promising results [383]. For example, experimental work with DNA and peptide-based vaccines in murine models has progressed, including a notable development involving a peptide vaccine derived from the HCV p7 protein [387]. Similarly, a DNA-based HCV vaccine has been effective in eliciting comprehensive T cell responses and memory, though it also stimulated a non-neutralizing antibody response [388]. The utility of messenger RNA (mRNA) vaccine technology, which gained momentum during the COVID-19 pandemic, represents a promising area to probe for HCV prevention [389]. However, significant challenges in the context of HCV vaccination remain, such as the extensive variability of HCV E proteins. Moreover, the low incidence of HCV in industrialized countries complicates conducting the clinical trials, which are often restricted to high-risk sub-populations. Nevertheless, ongoing research efforts, including a notable study by Patra et al., highlights the possibility of developing an mRNA vaccine platform to combat HCV effectively [390]. However, these efforts yielded promising early-stage vaccine candidates and simultaneously highlighted the need for ongoing research efforts to develop an HCV vaccine, which appears as a complex and lengthy journey [23]. 

## 14. Elimination of HCV by 2030

The WHO formulated a strategy aimed at mitigating the global health impact of hepatitis [391]. The WHO comprehensive hepatitis strategy targets a dramatic reduction in disease burden by 2030, endorsed universally by WHO member states. This strategy aims for a 90% reduction in new infections and a 65% decrease in mortality through an integrated approach that amplifies preventive measures such as TasP, expands the availability of diagnostic services, enhances access to antiviral therapies, and intensifies educational efforts [392,393]. 

In parallel, the Global HCV Elimination Coalition has implemented a multi-faceted approach to eradicate HCV by 2030 [394]. The revolutionary impact of DAAs is considered essential to these initiatives.

Additional strategies include the education and training of healthcare workers to ensure safe practices and effective patient education, which are essential for reducing new viral hepatitis cases, including HCV cases [395,396]. Integrating healthcare services to address the needs of marginalized populations, including IDUs, incarcerated individuals, and economically disadvantaged groups, is also important [397].

Together, these robust strategies necessitate that each country critically assess and adapt its healthcare frameworks to develop effective care to ensure comprehensive surveillance and management of hepatitis C cases [391]. This approach can reduce the incidence and mortality associated with hepatitis C and contribute to the ultimate goal of eliminating hepatitis C as a public health threat by 2030. 

## 15. Conclusions, Future Perspectives, and Limitations

In this comprehensive review of HCV, the remarkable advances and persistent challenges in diagnosis, management, and prevention of hepatitis C were highlighted. The development of DAAs revolutionized the management of chronic hepatitis C, significantly enhancing both treatment efficacy and prevention capabilities via TasP. However, the emergence of RASs remains a critical concern. This issue emphasizes the need for ongoing surveillance and the development of new pan-genotypic therapies that can effectively address the potentially resistant variants of HCV.

The absence of effective HCV vaccines so far continues to hinder efforts toward the WHO 2030 goal of HCV elimination. Achieving this ambitious goal will require enhanced diagnostic accessibility to ensure early and accurate detection, broader accessibility and enhanced affordability of DAAs, and improved adherence to treatment alongside robust monitoring of RAS impacts.

Continuous research and innovation are imperative to mitigate the global burden of HCV. Future research is needed to better predict and counteract the evolving dynamics of HCV transmission and resistance to DAAs. Coordinated global efforts are still needed to achieve the ultimate goal of HCV eradication or to at least alleviate its negative impact on public health.

Finally, we acknowledge several limitations inherent in this review despite its comprehensive nature, as follows. First, the selection bias is expected especially in terms of inclusion of studies published in English, with risk of missing significant findings reported in other languages. Second, the skewed availability of data towards more researched populations and regions might underrepresent the epidemiology especially in terms of absent estimates on the HCV prevalence in several countries worldwide. Lastly, we must acknowledge key limitations concerning the novelty and reproducibility of this review. Although we aimed to compile and analyze the current and past literature on HCV, the nature of this review did not involve the presentation of novel experimental findings but rather an attempt to integrate the existing knowledge on HCV to highlight trends and gaps in current HCV research.

## Figures and Tables

**Table 1:** The prevalence and total number of HCV infections in 2022 per country based on Polaris Observatory data [174].

Country	HCV Infections in 2022 (Prevalence %)	Country	HCV Infections in 2022 (Prevalence %)	Country	HCV Infections in 2022 (Prevalence %)	Country	HCV Infections in 2022 (Prevalence %)
Gabon	103,000 (4.31%)	Romania	186,000 (0.95%)	Thailand	375,000 (0.52%)	Türkiye	227,000 (0.27%)
Pakistan	9,859,000 (4.18%)	Albania	26,500 (0.94%)	Czechia	53,300 (0.51%)	Brazil	554,000 (0.26%)
Mongolia	131,000 (3.85%)	Rwanda	127,000 (0.92%)	Indonesia	1,381,000 (0.50%)	England	139,200 (0.25%)
Burundi	428,000 (3.32%)	Vietnam	905,000 (0.92%)	Cuba	53,700 (0.48%)	El Salvador	15,100 (0.24%)
Ukraine	1,290,000 (3.25%)	Benin	120,000 (0.90%)	Egypt	493,000 (0.44%)	Iran	211,000 (0.24%)
Uzbekistan	1,045,000 (3.02%)	Italy	526,000 (0.89%)	Croatia	17,800 (0.44%)	Afghanistan	97,710 (0.24%)
Tajikistan	256,000 (2.57%)	Greece	91,200 (0.88%)	DRC ^2^	432,000 (0.44%)	Jordan	26,400 (0.23%)
Kyrgyzstan	167,000 (2.52%)	Kenya	468,000 (0.87%)	South Africa	253,000 (0.42%)	Bhutan	1800 (0.23%)
Georgia	84,600 (2.26%)	Chad	143,000 (0.81%)	Australia	107,000 (0.41%)	Hong Kong	17,100 (0.23%)
Armenia	59,500 (2.14%)	Yemen	261,000 (0.77%)	India	5,562,000 (0.39%)	Germany	179,000 (0.21%)
Kazakhstan	392,000 (2.02%)	Uganda	354,000 (0.75%)	Madagascar	115,000 (0.39%)	Sweden	22,000 (0.21%)
Latvia	36,600 (1.98%)	Lithuania	20,400 (0.74%)	Japan	481,000 (0.39%)	Slovakia	11,700 (0.21%)
Azerbaijan	201,000 (1.94%)	Luxembourg	4800 (0.74%)	Poland	150,000 (0.38%)	Malta	1000 (0.19%)
Russia	2,683,000 (1.85%)	Mozambique	244,000 (0.74%)	Philippines	428,000 (0.37%)	Austria	16,400 (0.18%)
UAE ^1^	145,000 (1.54%)	United States	2,475,000 (0.73%)	Nepal	113,000 (0.37%)	Belgium	21,300 (0.18%)
Qatar	41,300 (1.53%)	Gambia	18,200 (0.67%)	Tunisia	45,000 (0.36%)	Republic of Korea	94,000 (0.18%)
Libya	98,200 (1.44%)	Argentina	301,000 (0.66%)	Portugal	37,400 (0.36%)	Peru	58,300 (0.17%)
Ghana	440,000 (1.31%)	Israel	58,800 (0.65%)	New Zealand	18,500 (0.36%)	Chile	32,200 (0.16%)
Syria	290,000 (1.31%)	Ecuador	115,000 (0.64%)	Switzerland	31,100 (0.36%)	France	100,000 (0.15%)
Puerto Rico	42,200 (1.30%)	Algeria	284,000 (0.63%)	Oman	15,700 (0.34%)	Tanzania	98,700 (0.15%)
Bulgaria	85,000 (1.25%)	Nigeria	1,352,000 (0.62%)	Iraq	151,000 (0.34%)	Slovenia	2700 (0.13%)
Burkina Faso	283,000 (1.25%)	Colombia	313,000 (0.60%)	Canada	130,000 (0.34%)	Lebanon	6300 (0.11%)
Cambodia	185,000 (1.10%)	Cameroon	166,000 (0.59%)	Morocco	125,000 (0.33%)	Denmark	5600 (0.10%)
Papua New Guinea	110,000 (1.08%)	Bangladesh	1,015,000 (0.59%)	Malaysia	110,000 (0.32%)	Saudi Arabia	31,800 (0.09%)
Bahrain	15,900 (1.08%)	Dominican Republic	65,400 (0.58%)	Panama	14,200 (0.32%)	Norway	4600 (0.08%)
Sudan	503,000 (1.07%)	Venezuela	164,000 (0.58%)	Finland	16,100 (0.29%)	Spain	39,600 (0.08%)
Estonia	14,100 (1.06%)	Ethiopia	694,000 (0.56%)	Hungary	28,800 (0.29%)	Netherlands	14,200 (0.08%)
Taiwan	251,000 (1.05%)	Mexico	684,000 (0.54%)	China	4,013,000 (0.28%)	Fiji	720 (0.08%)
Vanuatu	3400 (1.04%)	Ireland	26,800 (0.53%)	CAR ^3^	15,200 (0.27%)	Iceland	230 (0.06%)

^1^ UAE: United Arab Emirates; ^2^ DRC: Democratic Republic of the Congo; ^3^ CAR: Central African Republic.

**Table 2:** List of commonly used direct-acting antiviral (DAA) regimens.

Regimen (Trade Name)	Genotype/Subtype Coverage and Sustained Virologic Response (SVR)	Common Side Effects (>1%)
Glecaprevir/Pibrentasvir (Mavyret)	SVR12 of 99.7% for genotype 1 [331] SVR12 of 91% for genotype 3 [332] SVR12 of 99–100% for genotypes 4, 5, and 6 [333,334]	Headache and fatigue [331]
Sofosbuvir/Velpatasvir (Epclusa)	SVR12 of 98.1% for subtype 1a, 99.2% for subtype 1b, 100% for genotypes 2, 4, and 6, and 97.1% for genotype 5 [335] SVR12 of 95% for genotype 3 [336]	Headache, fatigue, nasopharyngitis, and nausea [335]
Ledipasvir/Sofosbuvir (Harvoni)	SVR12 of 95–98.6% for genotype 1 [337,338] SVR12 of 98.4% for genotype 2 [338] SVR12 of 98% for genotype 4 [339] SVR12 of 92.9% for genotype 5 [340] SVR12 of 95% for genotype 6 [341]	Fatigue, insomnia, headache, and nausea [340,341]
Elbasvir/Grazoprevir (Zepatier)	SVR12 of 92% for subtype 1a, 99% for subtype 1b, 100% for genotype 4, and 80% for genotype 6 [342]	Headache, fatigue, and nausea [342]

## Data Availability

The original contributions presented in the study are included in the article; further inquiries can be directed to the corresponding author (M.S.).
